# miR-126-3p Protects Neonatal Rats With Hypoxic-Ischemic Brain Damage Through Targeting LPR6 via PKC/ERK Signaling Pathway

**DOI:** 10.33549/physiolres.935510

**Published:** 2025-06-01

**Authors:** Wangsheng MA, Hui LI, Lin ZHA, Jingguo MA

**Affiliations:** 1 Pediatric Department, Puren Hospital Affiliated to Wuhan University of Science and Technology, Qingshan District, Wuhan, China

**Keywords:** miR-126-3p, LPR6, PKC/ERK pathway, Hypoxic-ischemic brain damage

## Abstract

Neonatal hypoxic-ischemic brain damage (HIBD) is a common factor in neonatal fatalities. miR-126-3p content in cerebral hemorrhage patients is obviously decreased, but its mechanism of action in HIBD is still unclear. The HIBD model was constructed by Rice-Vannucci method, and the change in miR-126-3p was detected. The target genes of miR-126-3p were obtained by database (miRWalk, TargetScan, miRTarbase and miRDB) analysis. The targeting relationship between miR-126-3p and low density lipoprotein receptor related protein (LRP6) was explored based on a dual luciferase assay. miR-126-3p over- and lowexpressed, LRP6 overexpressed and protein kinase C (PKC) pathway agonist phorbol 12-myristate 13-acetate (PMA) were injected into the brains of neonatal rats. The pathological changes in cerebral tissue and neuronal survival were observed by pathological staining. The neurological function was evaluated by foot fault test and wire suspension test. The levels of interleukin (IL)-1β, IL-6 and tumor necrosis factor-α (TNF-α) were tested by an ELISA kit. The levels of miR-126-3p, LRP6 and PKC/ERK pathway proteins were tested by qRT-PCR and Western blot. Knockdown of miR-126-3p can aggravate inflammation, brain tissue pathology and neurological impairment in HIBD, while miR-126-3p overexpression can improve it. miR-126-3p can target down-regulate LRP6. miR-126-3p can improve HIBD by down-regulating LRP6 expression and activating the PKC/ERK signaling pathway. miR-126-3p can target down-regulate LRP6 by activating the PKC/ERK signaling pathway to inhibit inflammation in HIBD rats, reduce brain tissue pathology and neurological damage, and improve HIBD.

## Introduction

Neonatal hypoxic-ischemic brain damage (HIBD) is a brain nerve dysfunction attributed to asphyxia and cerebral blood flow changes caused by various factors during the perinatal period [1]. It is mainly manifested as neonatal consciousness, muscle tension changes and primitive reflex abnormalities, which may cause neonatal death, epilepsy and other sequelae [2–4]. Studies have shown that 20–25 % of HIBD children die, and 25–30 % of survivors leave permanent neurological dysfunction and neurodevelopmental disorders [5], which is a common cause of neonatal morbidity and mortality [6]. Ischemia and hypoxia can reduce the neonatal brain blood, leading to acute failure of hippocampal neurons and neuronal death [7]. At present, the treatment of HIBD is mainly mild hypothermia treatment. In view of the short time window of mild hypothermia treatment in clinical practice, it cannot effectively repair damaged nerve cells. There are still a large number of newborns who die or have serious long-term neurological abnormalities [8]. Therefore, it is urgent to seek new treatments. With the continuous development of molecular biology and genomics, more and more targeted therapy strategies have been found, which brings new hope for the treatment of HIBD.

MicroRNA (miRNA) is a non-coding small RNA that regulates gene expression at the post-transcriptional level. The length is usually between 18–24 nucleotides. It can control gene activity, mainly by associating with downstream target gene mRNA to block its translation or degradation of mRNA, thereby inhibiting protein expression [9,10]. miRNAs are highly conserved in eukaryotes and control many important physiological and pathological processes [11]. Studies have found that 70 % of miRNAs are expressed in the central nervous system, and miRNAs have attracted more and more attention in central nervous regulation and related diseases [12,13]. miRNA may be vital in the regulation of the neuroinflammation response after brain injury [14]. At the same time, miR-126 is vital for maintaining vascular integrity and regulating angiogenic processes [15]. And miR-126-3p content in patients with intracerebral hemorrhage decreased obviously [16]. Therefore, miR-126-3p shows significant promise for treating HIBD, but there is no relevant report.

The protein kinase C (PKC)/extracellular signal-regulated kinase (ERK) pathway is crucial for regulating the cerebral nervous system, which is related to neurodevelopmental disorders and perinatal learning and memory disorders [17]. PKC/ERK pathway can improve oxidative stress and neuronal apoptosis [18,19]. In hypoxic neurons, the PKC/ERK pathway can protect neurons [20]. In addition, the PKC/ERK pathway in developing hippocampal neurons inhibits ketamine-induced early and late apoptosis in hippocampal neurons [21], but its impact on HIBD is unknown.

Therefore, we hypothesized that miR-126-3p and the PKC/ERK pathway are associated with HIBD pathogenesis. This research investigated the impact of miR-126-3p on HIBD through HIBD rat model, screened the target genes and potential regulatory pathways of miR-126-3p, and analyzed the impact of this pathway on neuroinflammation and neurological function in order to provide new ideas and a theoretical basis for future molecular targeted therapy of HIBD based on miR-126-3p.

## Methods

### Animal grouping and processing

A total of 128 7-day-old healthy Sprague-Dawley (SD) neonatal rats, weighing (12±2) g, male or female, were provided by Spyford Biotechnology Co., Ltd. (Beijing, China). Rats were fed under normal conditions (temperature 20–24 °C, relative humidity 50–70 %, light and dark 12/12 h). This experiment was approved by Puren Hospital affiliated with the Wuhan University of Science and Technology Animal Ethics Committee.

All rats were randomly divided into sham operation (Sham) group, HIBD model (HI) group, miR-126-3p inhibitors negative control + HIBD (i-NC+HI) group, miR-126-3p inhibitors + HIBD (i+HI) group, miR-126-3p mimics negative control + HIBD (m-NC+HI) group, miR-126-3p mimics + HIBD (m+HI) group, miR-126-3p mimics negative control + recombinant low density lipoprotein receptor related protein 6 (LRP6) mimics negative control + HIBD (m-NC+OE-NC+HI) group, miR-126-3p mimics + LRP6 mimics negative control + HIBD (m+OE-NC+HI) group, miR-126-3p mimics + LRP6 mimics + HIBD (m+OE-LRP6+HI) group and miR-126-3p mimics + LRP6 mimics + PKC activator phorbol 12-myristate 13-acetate (PMA) + HIBD (m+OE-LRP6+PMA+HI) group, 8 rats in each group. According to the 4 time points of 3, 6, 12 and 24 h after hypoxic-ischemic, 8 rats were randomly selected from the Sham group and the HI group at each time point for the study.

The rats were sedated through inhalation of 2–3 % isoflurane. The head of the rats was fixed with a stereotactic syringe (EZ Scientific, Yizejia Technology Co., Ltd., Beijing, China), and the skull was exposed after disinfecting the skin of the head. According to the stereotactic map of the rats [22], the skull was drilled at 1 mm to the right of the middle line of the right brain and 0.5 mm behind the sagittal line. 2 μl of corresponding solution (50 pmol/μl miR-126-3p mimics NC, 50 pmol/μl miR-126-3p mimics, 50 pmol/μl miR-126-3p inhibitors NC, 50 pmol/μl miR-126-3p inhibitors, 50 pmol/μl LRP6 mimics NC, 50 pmol/μl LRP6 mimics, 2 μg/kg PKC acti-vator PMA) was drawn into 3 mm using a microsyringe. The injection speed was controlled at 1 μl/min, and the lateral ventricle of the ipsilateral hemisphere of the rats in the corresponding group was injected. After the injection, the needle was stopped for 5 min and the syringe was slowly removed. Then, the small holes were closed with bone wax, and then the rats continued to be fed. After 48 h, the HIBD model was constructed by the modified Rice-Vannucci method [23]. After anesthesia in rats, the right common carotid artery was exposed through the median cervical incision, and the incision was sutured after double-line ligation. After the operation, the newborn rats were recovered in a 37 °C incubator for 1 h, and then placed in an anoxic box (92 % N_2_ + 8 % O_2_) for 20 min. Finally, they were placed back to the mother rats and reoxygenated in a normal environment. The Sham group was not ligated and did not give hypoxia treatment. The specific grouping and operations are shown in Table 1.

### Bioinformatics analysis

The potential genes of miR-126-3p were obtained by TargetScan (https://www.targetscan.org/vert_80/), miRTarbase (http://mirtarbase.mbc.nctu.edu.tw/index.html), miRDB (http://www.mirdb.org/), and miRWalk (http://129.206.7.150/) databases. TargetScan predicted the binding sites of miR-126-3p and LRP6.

### Double luciferase assay

The 3′-UTR fragment of the potential binding site of miR-126-3p and LRP6 was cloned into the plasmid vector for transformation. Plasmids with miR-126-3p and LRP6 binding sites were designed on wild-type (WT) and mutant (MUT) sites. Following Lipofectamine 2000 (Invitrogen Inc., Carlsbad, CA, USA) kit, plasmid 200 ng and 30 nM miR-126-3p mimics or mimics NC were extracted and co-transfected at 37 °C for 24 h. The fluorescence intensity was tested using a dual luciferase detection system (Promega Corporation, Madison, WI, USA).

### Nerve injury assessment

After 14 days of injury, neurological impairment was evaluated. Following the method of Horiquini *et al*. [24], the foot fault test was carried out, the number of foot failures of forelimbs and hindlimbs was recorded, and the failure rate was calculated. The wire suspension test was performed using the method of Ahmed *et al*. [25] to test the forelimb strength.

### ELISA assay

Interleukin-6 (IL-6, SEKR-0005), interleukin-1β (IL-1β, SEKR-0002), tumor necrosis factor-α (TNF-α, SEKR-0009) ELISA kits were obtained from Solarbio (Beijing, China). After the experiment, the rat brain tissue was fully homogenized on ice, and the supernatant was gathered. IL-6, IL-1β and TNF-α were tested following the operation steps.

### HE staining

The rat brain tissue was taken, fixed with 4 % paraformaldehyde, embedded in paraffin, and then the tissue specimen was cut into 4 μm thick sections. The sections were stained with hematoxylin (C0107, Beyotime, Shanghai, China) for 15 min, differentiated in 1 % acidic alcohol for 30 s, rinsed with running water, and then stained with 0.5 % eosin (G1100, Solarbio) for 3 min. Then the slices were dehydrated by alcohol gradient dehydration and xylene transparent treatment, neutral gum (G8590, Solarbio) sealing, and the morphology of cerebral cortex and hippocampal C1 region neurons were observed under a microscope.

### Nissl staining

Nissl bodies were stained by Nissl staining kit (G1434, Solarbio). The sections of brain tissue were stained with methylene blue for 10 min, and placed in differentiation medium for 1 min, treated with ammonium molybdate solution for 3 min, washing with distilled water to prevent decolorization, and mounting with neutral gum (G8590, Solarbio). The number and morphological changes of Nissl bodies in the cerebral cortex and hippocampal C1 region were observed under a microscope.

### Fluoro Jade C staining

Fluoro Jade C (FJC) kit (TR-100-FJT, Biosensis, US) was used to detect the degeneration of hippocampal neurons. After routine dewaxing and hydration, the paraffin sections of brain tissue were immersed in potassium permanganate solution for 10 min, then stained in FJC solution for 10 min, rinsed with distilled water, dehydrated with anhydrous ethanol, transparentized with xylene for 5 min, and sealed with neutral gum. The changes in the number of degenerative neurons in the cerebral cortex and hippocampal C1 region were observed under a microscope.

### Immunofluorescence

The brain sections of each group were dewaxed with xylene, hydrated, and antigen repaired. The sections were blocked with 5 % bovine serum albumin (BSA) and 0.5 % Triton-X100 mixed solution for 2 h. Rabbit anti-neuronal nuclei (NeuN, ab177487, 1:1000, Abcam) were added and incubated overnight at 4 °C, followed by incubation with fluorescent secondary antibody goat anti-rat IgG (GB21302, 1:500, Servicebio, Wuhan, China) for 2 h. The tablet (S2110, Solarbio) was sealed, and fluorescence images were obtained using a fluorescence microscope (Olympus, VS200, Shinjuku, Tokyo) to observe the survival of neurons in the cerebral cortex and hippocampal C1 region.

### TUNEL staining

The apoptosis in cerebral cortex and hippocampus was tested by Tunel kit (C1091, Beyotime). The sections were permeabilized with 20 μg/ml protease K solution without deoxyribonuclease (DNase) for 30 min. Apoptotic cells were labeled with 50 μl Tunel solution and incubated in dark for 60 min. 4′,6-DiAmidino-2-PhenylIndole (DAPI) staining solution was employed to stain the nucleus for 5 min. The sections were sealed by an anti-fluorescence quenching solution, and Tunel positive in the cerebral cortex and hippocampal C1 region was observed by a fluorescence microscope.

### qRT-PCR

RNA was abstracted by TransZol Up (ET111-01-V2, TRANS, Beijing, China), and then AMV reverse transcriptase (2621, TAKARA, Tokyo, Japan) was supplied for reverse transcription to obtain cDNA. Then TB Green FAST qPCR (CN830S, TAKARA) was used for the PCR reaction. The relative level of mRNA was computed by the 2^−ΔΔCt^ method. U6 and GAPDH can serve as controls.

The primer sequences: miR-126-3p: F: 5′-TCTTCTGTGGGTGGACACTG-3′; R: 5′-CTTCAT-CTTCACCTTGTGGA-3′; LRP6: F: 5′-CTGAATGCT-GACAACAGGACCTG-3′; R: 5′-GACGTTCCGAAG-GCTGTGGATA-3′; U6: F: 5′-CTCGCTTCGGC-AGCACAT-3′; R: 5′-TTTGCGTGTCATCCTTGCG-3′; GAPDH: F: 5′-CATCACTGCCACCCAGAAGACTG-3′; R: 5′-ATGCCAGTGAGCTTCCCGTTCAG-3′.

### Western blot

After the brain tissue was collected, the samples were fully lysed by RIPA lysis buffer. The protein concentration was tested using BCA kit (PC0020, Solarbio). All samples were detached by electrophoresis, and the protein was then transferred to the polyvinylidene fluoride (PVDF) membranes (YA1700, Solarbio). The membranes were incubated with 5 % skimmed milk powder (LP0033B, Solarbio) for 2 h, and LRP6 (ab134146, 1:1000, Abcam), PKC (ab181558, 1:2000, Abcam), p-PKC (ab109539, 1:1000, Abcam), ERK1/2 (ab184699, 1:10000, Abcam), p-ERK1/2 (ab201015, 1:1000, Abcam) and glyceraldehyde-3-phosphate dehydrogenase (GAPDH, TA-08, 1:1000, ZSGB-BIO, Beijing, China) incubated them at 4 °C for one night. On the next day, the membranes were washed with Tris-buffered saline with Tween-20 (TBST) buffer (T1082, Solarbio) and incubated with secondary antibody (1:20000) for 1 h. After 5 times washed with TBST buffer, enhanced chemiluminescence (ECL, PE0010, Solarbio) reagent was used to react for 2–3 min, and then automatic chemiluminescence imaging system was used for imaging.

### Statistical analysis

Each experiment was repeated at least 3 times, and all data were expressed as mean ± standard deviation. Statistical analysis and image drawing were performed using GraphPad 9.0. Student’s *t*-test was used to compare the difference between two groups, and One-way ANOVA analysis was used to compare multiple groups. *P*<0.05 was considered statistically significant.

## Results

### The hypoxic-ischemic brain damage (HIBD) model of neonatal rats was constructed, and miR-126-3p was lowered in HIBD rats

**Fig. 1:**
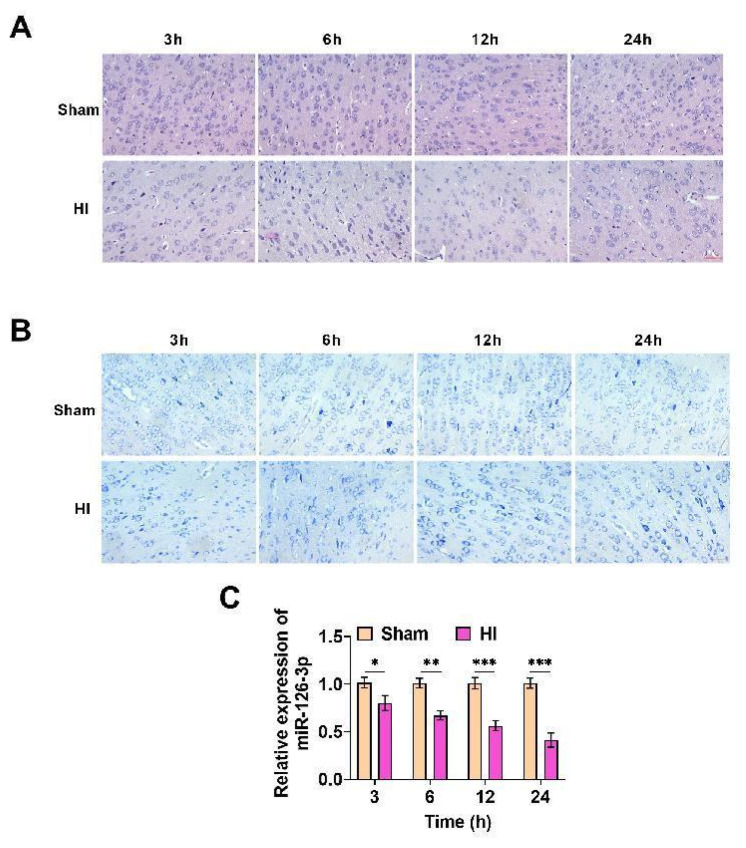
The hypoxic-ischemic brain damage (HIBD) model of neonatal rats was constructed, and miR-126-3p was lowered in HIBD rats. (**A**) HE staining showed that the neurons were swollen and irregular in shape in HIBD, and became more and more serious with the increase of ischemia and hypoxia time. (**B**) Nissl staining showed that the morphology of Nissl bodies was disordered in HIBD and became more serious with the increase of ischemia and hypoxia time. (**C**) miR-126-3p in brain tissue was tested, it was significantly down-regulated in HIBD, and the down-regulation was more obvious with the increase of ischemia and hypoxia time (*P*<0.05, *P*<0.01, *P*<0.001).

Neuronal apoptosis, neuronal dysfunction and structural abnormalities are important pathological processes of HIBD [26]. In order to verify whether the HIBD model was successfully constructed, we evaluated brain tissue damage by HE and Nissl staining. The shape of the cerebral cortex neurons of normal rats was standard, and the arrangement was regular (Fig. 1A). The structure of Nissl bodies was intact, showing blue particles or plaques (Fig. 1B). In the HI group, the nerve cells in the cerebral cortex were swollen and irregular, and the Nissl bodies were disordered. With the increase of ischemia and hypoxia time, the damage of nerve cells was more serious, which indicated that the phenomenon of nerve tissue damage occurred, indicating that the HIBD model was effectively constructed. Next, miR-126-3p was notably lowered in HI group at each time period, and the down-regulation was more obvious with the increase of ischemia and hypoxia time (Fig. 1C), suggesting the potential protective effect of miR-126-3p in HIBD.

### Knockdown of miR-126-3p increases the level of pro-inflammatory cytokines in neonatal rats with hypoxic-ischemic brain damage (HIBD) and aggravates HIBD in neonatal rats

**Fig. 2:**
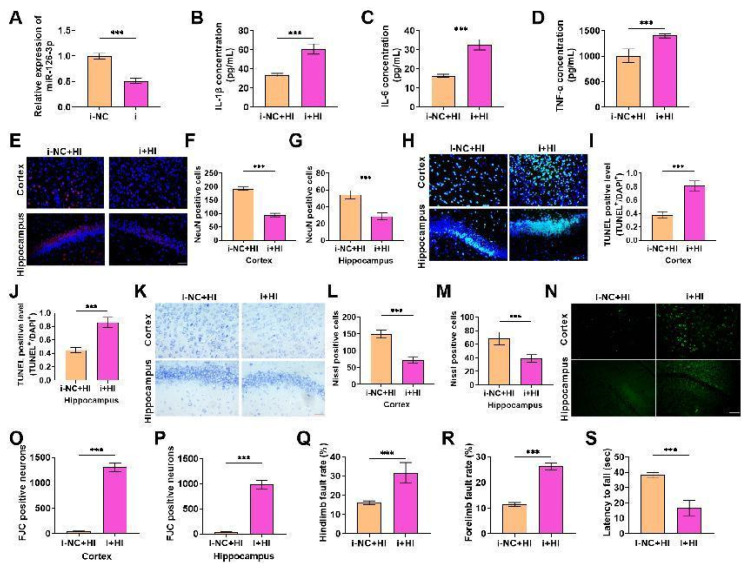
Knockdown of miR-126-3p increases the level of pro-inflammatory cytokines in neonatal rats with hypoxic-ischemic brain damage (HIBD) and aggravates HIBD in neonatal rats. (**A**) miR-126-3p inhibitors were injected into the brains of rats, and the knockdown efficiency was tested, it was effectively declined (*P*<0.001). (**B–D**) The levels of IL-1β, IL-6 and TNF-α were notably increased after miR-126-3p knockdown by ELISA kits (*P*<0.001). (**E–G**) The survival rate of neurons was tested by immunofluorescence, and the survival rate was notably diminished after miR-126-3p knockdown (*P*<0.001). (**H–J**) The apoptotic cells in cerebral neurons were tested by TUNEL staining. The apoptotic cells increased significantly after miR-126-3p knockdown (*P*<0.001). (**K–M**) The neurons were tested by Nissl staining, and the number of neurons was notably declined after miR-126-3p knockdown (*P*<0.001). (**N–P**) The number of FJC positive cells was tested by FJC staining. It was significantly increased after miR-126-3p knockdown (*P*<0.001). (**Q–R**) Foot fault test showed that miR-126-3p knockdown notably elevated the rate of forelimb and hindlimb fault in rats (*P*<0.001). (**S**) The wire suspension test showed that the forelimb strength decreased significantly after miR-126-3p knockdown (*P*<0.001).

To explore the function of miR-126-3p in HIBD, we injected miR-126-3p inhibitors into the brain by stereotactic injection. We first detected its knockdown efficiency, and it was declined (Fig. 2A), suggesting that it was effectively knocked down, and subsequent experiments could be carried out. Inflammatory response is one of the manifestations of HIBD [27]. Therefore, we detected the inflammatory indexes. The contents of IL-1β, IL-6 and TNF-α were evidently enhanced after knockdown (Fig. 2B–D), indicating that miR-126-3p knockdown could aggravate the inflammatory response of HIBD in neonatal rats. After that, the survival rate of neurons of the i+HI group was obviously declined (Fig. 2E–G), the apoptosis was significantly elevated (Fig. 2H–J), the number of Nissl staining positive cells was also significantly reduced (Fig. 2K–M), and the number of green-stained FJC positive cells was significantly increased (Fig. 2N–P), indicating that miR-126-3p knockdown can further damage the brain tissue and nerve function of rats. Foot fault test and wire suspension test also showed this result. The forelimb and hindlimb fault rates of rats in the i+HI group increased significantly (Fig. 2Q–R), and the forelimb strength decreased significantly (Fig. 2S). In conclusion, miR-126-3p knockdown can damage the neurological function of brain tissue by aggravating the inflammatory response, thus promoting the progress of HIBD in neonatal rats, so miR-126-3p can treat HIBD.

### Overexpression of miR-126-3p reduces the level of pro-inflammatory cytokines in neonatal rats with hypoxic-ischemic brain damage (HIBD) and alleviates HIBD in neonatal rats

**Fig. 3:**
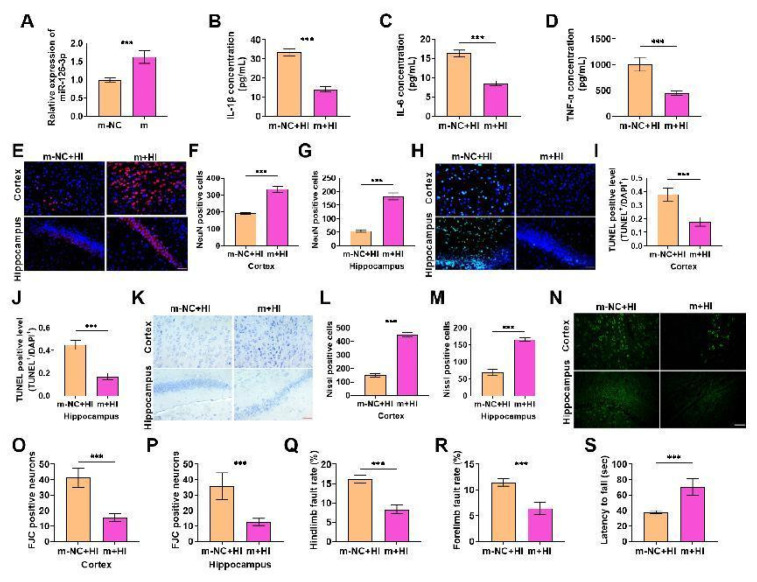
Overexpression of miR-126-3p reduces the level of pro-inflammatory cytokines in neonatal rats with hypoxic-ischemic brain damage (HIBD) and alleviates HIBD in neonatal rats. (**A**) miR-126-3p mimics were injected into the brains of rats, and the overexpression efficiency was tested, it was effectively overexpressed (*P*<0.001). (**B–D**) ELISA showed that the contents of inflammatory factors were notably declined after miR-126-3p overexpression (*P*<0.001). (**E–G**) The survival rate of cerebral neurons was detected by immunofluorescence, and the survival rate was markedly increased after miR-126-3p overexpression (*P*<0.001). (**H–J**) The apoptotic cells were tested by TUNEL staining. The number of apoptotic cells was markedly shrunk after miR-126-3p overexpression (*P*<0.001). (**K–M**) The number of neurons was detected by Nissl staining, and it was notably increased after miR-126-3p overexpression (*P*<0.001). (**N–P**) The number of FJC positive cells was tested by FJC staining. It was significantly decreased after miR-126-3p overexpression (*P*<0.001). (**Q–R**) The foot fault test showed that the rate of forelimb and hindlimb foot fault in rats was notably shrunk after miR-126-3p overexpression (*P*<0.001). (**S**) The wire suspension test showed that the forelimb strength enhanced significantly after miR-126-3p overexpression (*P*<0.001).

Next, we overexpressed miR-126-3p, and detected the overexpression efficiency, and it was effectively highly expressed (Fig. 3A), suggesting that subsequent experiments can be carried out. Then we detected the inflammatory markers by ELISA kit, and the contents of inflammatory factors were notably decreased after overexpression (Fig. 3B–D), indicating that overexpressed miR-126-3p can inhibit the inflammation of HIBD in neonatal rats. After that, the survival rate of cerebral cortex and hippocampal neurons was notably enhanced (Fig. 3E–G), the apoptosis was markedly reduced (Fig. 3H–J), the number of Nissl staining positive cells was also significantly increased after overexpression (Fig. 3K–M), and the number of FJC positive cells was significantly reduced (Fig. 3N–P), indicating that overexpressed miR-126-3p can markedly inhibit brain tissue and neurological damage in HIBD rats. Foot fault test and wire suspension test also showed this result. The forelimb and hindlimb fault rate of rats in i+HI group was significantly reduced (Fig. 3Q–R), and the forelimb strength was significantly increased (Fig. 3S). In short, overexpressed miR-126-3p can improve the neurological function of brain tissue by inhibiting inflammatory response, thereby reducing HIBD in neonatal rats. At the same time, combined with the results of knockdown experiments, the low expression of miR-126-3p can aggravate HIBD, but its overexpression can inhibit HIBD, which together indicate that miR-126-3p has a protective function and is a key regulator of HIBD.

### miR-126-3p can target low density lipoprotein receptor related protein 6 (LRP6) and down-regulate LRP6 expression

**Fig. 4:**
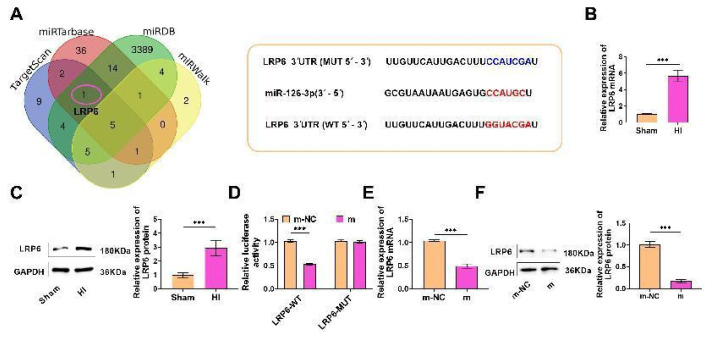
miR-126-3p can target low density lipoprotein receptor related protein 6 (LRP6) and down-regulate LRP6 expression. (**A**) The potential target gene LRP6 of miR-126-3p was obtained. The combining sites of miR-126-3p and LRP6 were predicted by TargetScan database, indicating that miR-126-3p could bind to LRP6. (**B**) LRP6 mRNA was tested, and which in HI group was evidently enhanced (*P*<0.001). (**C**) LRP6 protein was tested, and in HI group was evidently enhanced (*P*<0.001). (**D**) LRP6-WT and LRP6-MUT luciferase reporter plasmids were co-transfected with miR-126-3p mimics NC and mimics, respectively, and luciferase activity was tested. Overexpressed miR-126-3p obviously inhibited the luciferase activity of LRP6-WT. (**E**) LRP6 mRNA was tested after overexpression of miR-126-3p, and LRP6 mRNA was notably declined (*P*<0.001). (**F**) LRP6 protein was tested by Western blot after miR-126-3p overexpression, and it was notably declined (*P*<0.001).

Bioinformatics showed that the potential target of miR-126-3p was LRP6, and there were potential combining sites. The binding sequence was shown in Fig. 4A, so miR-126-3p could target LRP6. Next, we detected the LRP6 mRNA and protein levels. LRP6 levels in HI group were notably increased (Fig. 4B–C), suggesting that LRP6 has a potential role in promoting HIBD, indicating that LRP6 may be a key factor in HIBD. Next, we used luciferase reporter gene experiments to verify whether miR-126-3p binds to LRP6. miR-126-3p overexpression notably inhibited the luciferase activity of LRP6-WT (Fig. 4D). At the same time, LRP6 mRNA and protein were notably declined after overexpression (Fig. 4E–F), further demonstrating that miR-126-3p binds to LRP6. In conclusion, miR-126-3p targetedly down-regulated LRP6 at the post-transcriptional level.

### Low density lipoprotein receptor related protein 6 (LRP6) overexpression attenuated the phenomenon that miR-126-3p overexpression reduced hypoxic-ischemic brain damage (HIBD) in neonatal rats

**Fig. 5:**
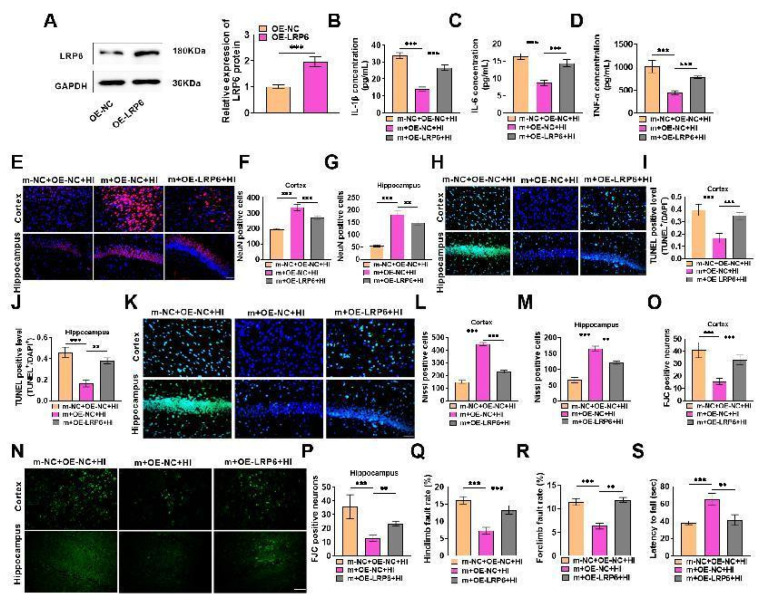
Low density lipoprotein receptor related protein 6 (LRP6) overexpression attenuated the phenomenon that miR-126-3p overexpression reduced hypoxic-ischemic brain damage (HIBD) in neonatal rats. (**A**) LRP6 mimics were injected into the brain, and the overexpression efficiency of LRP6 in brain tissue was tested, it was effectively overexpressed (*P*<0.001). (**B–D**) The levels of inflammatory factors were notably enhanced after LRP6 overexpression compared with miR-126-3p overexpression alone (*P*<0.001). (**E–G**) The survival rate of cerebral cortex and hippocampal neurons was detected by immunofluorescence. The survival rate of LRP6 overexpression was markedly lowered (*P*<0.001, *P*<0.01). (**H–J**) The apoptotic cells were tested by TUNEL staining. After overexpression of LRP6, the apoptotic cell number was notably raised (*P*<0.001, *P*<0.01). (**K–M**) The number of neurons was tested by Nissl staining, it was notably shrunk after LRP6 overexpression (*P*<0.001, *P*<0.01). (**N–P**) The number of FJC positive cells was tested by FJC staining. It was significantly increased after LRP6 overexpression (*P*<0.01, *P*<0.001). (**Q–R**) Foot fault test showed that compared with miR-126-3p overexpression alone, LRP6 overexpression markedly elevated the rate of forelimb and hindlimb fault in rats (*P*<0.001, *P*<0.01). (**S**) Wire suspension test showed that LRP6 overexpression significantly reduced forelimb strength compared with miR-126-3p overexpression alone (*P*<0.001, *P*<0.01).

We further explored whether miR-126-3p can target LRP6 to affect HIBD. We first verified the efficiency of LRP6 overexpression, and LRP6 protein was effectively overexpressed (Fig. 5A), suggesting that subsequent experiments can be performed. Then the experiment was divided into HIBD model (m-NC+OE-NC+HI) group, miR-126-3p overexpression (m+OE-NC+HI) group, miR-126-3p overexpression + LRP6 overexpression (m+OE-LRP6+HI) group. Similar to the results of 3.3, overexpressed miR-126-3p reduced pro-inflammatory cytokine levels in neonatal rats with HIBD and inhibited HIBD; On this basis, LRP6 was overexpressed, the contents of inflammatory factors were notably enhanced (Fig. 5B–D), the survival rate of neurons was significantly declined (Fig. 5E–G), the apoptosis was markedly raised (Fig. 5H–J), the number of Nissl staining positive cells was also significantly decreased (Fig. 5K–M), the number of FJC positive cells was significantly increased (Fig. 5N–P), the rate of forelimb and hindlimb fault was significantly increased (Fig. 5Q–R), and the forelimb strength was significantly decreased (Fig. 5S). It is indicated that overexpression of LRP6 can further stimulate the inflammatory response of HIBD in neonatal rats, damage brain tissue and nerve function, and aggravate HIBD. In short, overexpressed miR-126-3p can inhibit HIBD, but on this basis, overexpression of LRP6 markedly weakens the improvement effect of miR-126-3p overexpression on HIBD. Joint bioinformatics results, miR-126-3p can target down-regulate LRP6 to reduce HIBD in neonatal rats.

### miR-126-3p inhibits hypoxic-ischemic brain damage (HIBD) in neonatal rats by targeting low density lipoprotein receptor related protein 6 (LRP6) through the PKC/ERK pathway

**Fig. 6:**
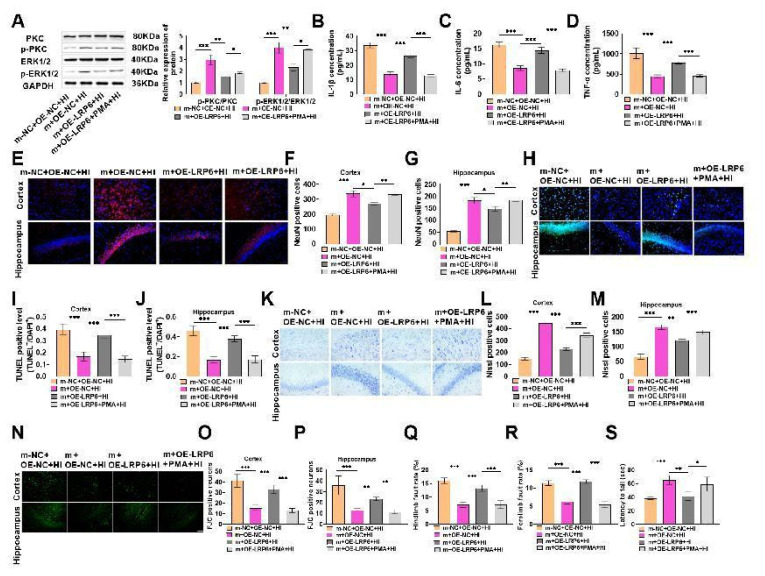
miR-126-3p inhibits hypoxic-ischemic brain damage (HIBD) in neonatal rats by targeting low density lipoprotein receptor related protein 6 (LRP6) through the PKC/ERK signaling pathway. (**A**) PKC activator PMA was injected into the brains of rats, and PKC/ERK signaling pathway protein levels were detected. It was found that the p-PKC and p-ERK1/2 protein levels were notably elevated after overexpressed miR-126-3p (*P*<0.001). There were notably declined after miR-126-3p and LRP6 overexpression (*P*<0.01). On this basis, they were obviously elevated by PMA (*P*<0.05). (**B–D**) The levels of inflammatory factors were evidently decreased after PMA compared with miR-126-3p and LRP6 overexpression (*P*<0.001). (**E–G**) The survival rate of neurons was tested by immunofluorescence. After the application of PMA, the survival rate markedly rebounded (*P*<0.05, *P*<0.01, *P*<0.001). (**H–J**) The apoptotic cells were tested by TUNEL staining. After the application of PMA, the apoptotic cells were markedly reduced (*P*<0.001). K–M: The number of neurons was tested by Nissl staining. After the application of PMA, it was notably elevated (*P*<0.001). (**N–P**) The number of FJC positive cells was tested by FJC staining. After the application of PMA, it was significantly decreased (*P*<0.001, *P*<0.01). (**Q–R**) The foot fault test showed that compared with miR-126-3p and LRP6 overexpression, the rate of forelimb and hindlimb fault in rats was significantly reduced after PMA application (*P*<0.001). (**S**) The wire suspension test showed that compared with miR-126-3p and LRP6 overexpression, the forelimb strength increased significantly after PMA application (*P*<0.05, *P*<0.01, *P*<0.001).

To verify whether miR-126-3p can affect HIBD through PKC/ERK signaling pathway, the experiment was divided into HIBD model (m-NC+OE-NC+HI) group, miR-126-3p overexpression (m+OE-NC+HI) group, miR-126-3p overexpression + LRP6 overexpression (m+OE-LRP6+HI) group, miR-126-3p overexpression + LRP6 overexpression + PKC activator PMA (m+OE-LRP6+PMA+HI) group. p-PKC and p-ERK1/2 protein were markedly enhanced after miR-126-3p overexpression, indicating that overexpressed miR-126-3p can activate the PKC/ERK pathway, and miR-126-3p can adjust the PKC/ERK signaling pathway; when both miR-126-3p and LRP6 were overexpressed, p-PKC and p-ERK1/2 protein up-regulation was weakened, but there were significantly elevated after the application of PMA on this basis (Fig. 6A), indicating that LRP6 overexpression could weaken the activation of PKC/ERK signaling pathway caused by miR-126-3p overexpression, but PKC activator could re-activate PKC/ERK pathway on the basis of LRP6 overexpression. It is indicated that miR-126-3p can target LRP6 to trigger PKC/ERK pathway. In short, miR-126-3p can regulate PKC/ERK pathway by targeting LRP6.

To confirm the effect of PKC/ERK signaling pathway on HIBD, we found that compared with m+OE-LRP6+HI group, the contents of inflammatory factors in m+OE-LRP6+PMA+HI group were notably reduced (Fig. 6B–D), the survival rate of cerebral cortex and hippocampal neurons was significantly raised (Fig. 6E–G), the apoptosis was significantly decreased (Fig.6H–J), the number of Nissl staining positive cells was also significantly increased (Fig. 6K–M), the number of FJC positive cells was significantly decreased (Fig. 6N–P), and the rate of forelimb and hindlimb fault was significantly decreased (Fig. 6Q–R). The forelimb strength was significantly increased (Fig. 6S), indicating that PKC activator could improve the promoting effect of LRP6 overexpression on HIBD in neonatal rats, reduce the inflammatory response of HIBD, improve brain tissue and nerve function, and inhibit the progress of HIBD. Combined with the experimental results of PKC/ERK signaling pathway, miR-126-3p can target LRP6 to adjust PKC/ERK signaling pathway, which indicates that miR-126-3p targets LRP6 to adjust PKC/ERK signaling pathway, thereby inhibiting the inflammation of HIBD in neonatal rats and improving brain tissue and nerve function damage. In conclusion, miR-126-3p can improve HIBD by targeting LRP6 through PKC/ERK signaling pathway.

## Discussion

HIBD caused by hypoxia and disruption of cerebral blood supply is a factor in neonatal death [4]. HIBD treatment principle is to reconstruct neurological function and prevent the occurrence and development of brain injury. At present, the neuroprotective strategy, neurorehabilitation technology and related basic research on HIBD have made great progress, but there is no definite treatment for HIBD [28]. Studies have shown that it can play a neuroprotective role in HIBD by affecting neurons in the cerebral cortex and hippocampus. miR-126-3p is a microRNA involved in vascular integrity and inflammation. It is significantly reduced in cerebral hemorrhage patients and can diminish blood-brain barrier damage and neuronal damage around the hematoma after cerebral hemorrhage [16,29]. At the same time, miR-126-3p can also improve stroke by restoring the blood-brain barrier [30]. This suggests that miR-126-3p holds significant impact in brain injury diseases, but whether it can exert a protective effect on neonatal HIBD by affecting neurons in the cerebral cortex and hippocampus has not been reported. So, in this study, the HIBD model of neonatal rats was established by Rice-Vannucci method, and the effects and mechanisms of miR-126-3p and its potential targets on neurons in cerebral cortex and hippocampus after HIBD in neonatal rats were investigated.

The neurons after modeling were swollen and disordered, and the morphology of Nissl bodies was irregular, suggesting that the HIBD model was successfully created. miR-126-3p was notably diminished in HIBD, suggesting the potential protective effect of miR-126-3p in HIBD. miR-126 is strongly expressed in vascular-rich tissues [31] and has a positive regulatory effect in the angiogenesis pathway [32]. Mature miR-126 produces miR-126-3p. miR-126-3p can promote cardiac angiogenesis in rats [33], promote angiogenesis, inhibit inflammation, and weaken the destruction of the blood-brain barrier [29,34,35]. At the same time, miR-126-3p is also considered to be an anti-inflammatory miRNA [36]. Therefore, miR-126-3p has a great impact in HIBD, but its specific role and potential mechanism are not very clear, so it is discussed. We overexpressed/knocked down miR-126-3p, and miR-126-3p can reduce/increase pro-inflammatory factor levels, increase/decrease the survival rate of neurons and the rate of forelimb and hindlimb fault, and reduce/increase apoptosis and forelimb strength during wire suspension. In conclusion, miR-126-3p can inhibit/promote HIBD by inhibiting/promoting inflammation and improving/damaging brain tissue and nerve function, indicating that miR-126-3p can regulate pathological processes and has a protective effect on HIBD.

In this study, the potential target of miR-126-3p was LRP6 by bioinformatics, and further found that there was a potential combining site between them, so miR-126-3p could target LRP6. LRP6 levels were significantly enhanced in HIBD but declined notably after miR-126-3p overexpression, which further proved that miR-126-3p binds to LRP6, indicating that miR-126-3p targeted down-regulates LRP6. LRP6 belongs to the low density lipoprotein receptor superfamily [37] and engages in regulating various disease processes, including ischemic diseases [38]. LRP6 is highly expressed in neurons after cerebral hemorrhage [39]; it is also crucial in neuronal synaptic integrity and neuronal activity [40]. However, whether miR-126-3p can alleviate HIBD by targeting LRP6 is unknown. Therefore, we overexpressed LRP6 after miR-126-3p overexpression. The pro-inflammatory factors in neonatal rats with HIBD were significantly elevated, and the damage to brain tissue and nerve function was aggravated, indicating that LRP6 overexpression significantly weakened the improvement of miR-126-3p overexpression on HIBD. Combined with bioinformatics results, miR-126-3p can target down-regulate LRP6 to reduce HIBD in neonatal rats.

PKC is composed of the phospholipid-dependent serine/threonine kinase family, which is triggered with the second messenger diacylglycerol in a Ca^2+^-dependent manner. It is not only engaged in regulating various cell functions [41], but also the main regulator of the pro-inflammatory signaling center associated with various pathways’ transduction, including nuclear factor kappa-B (NF-κB) and mitogen-activated protein kinase (MAPK) pathways [42]. ERK1/2 is one of the main kinases of MAPK signaling pathway in mammalian cells, which is engaged in cell differentiation and apoptosis [43]. The activation of PKC can mediate the phosphorylation of ERK1/2 [44]. The PKC pathway is engaged in the neuroprotective effect by triggering ERK1/2 in focal cerebral hemorrhage [45–47]. In addition, PKC can regulate the transcriptional activity of ERK1/2 to reduce apoptosis [48]. However, whether miR-126-3p can affect HIBD through the PKC/ERK signaling pathway is unknown. Therefore, in this study, we verified by PKC activator PMA. The results showed that p-PKC and p-ERK1/2 were notably enhanced after miR-126-3p overexpression; on this basis, the protein up-regulation levels were weakened after LRP6 overexpression; then the protein levels were significantly raised after the application of PMA, indicating that miR-126-3p can target LRP6 to control the PKC/ERK pathway. Especially after the application of PMA, the inflammation and neurological impairment of HIBD in neonatal rats were significantly improved, indicating that PKC activator could improve the promoting effect of LRP6 overexpression on HIBD in neonatal rats. Combined with the experimental results of PKC/ERK signaling pathway, it is indicated that miR-126-3p can target LRP6 to prevent HIBD through PKC/ERK pathway.

## Conclusions

This article reveals the mechanism of miR-126-3p in HIBD of neonatal rats. miR-126-3p can inhibit pro-inflammatory factors, increase the survival rate and number of neurons, increase the rate of forelimb and hindlimb fault, reduce apoptosis and forelimb strength, and protect brain tissue and nerve function. It is confirmed that miR-126-3p has a protective impact on HIBD in neonatal rats and activates the PKC/ERK signaling pathway by down-regulating LRP6. This study provides a reliable reference for the screening of targeted therapy for HIBD, and miR-126-3p is a potential target for HIBD research and may play a key role in HIBD treatment. However, there are still some limitations, and the effectiveness of miR-126-3p in clinical application needs to be further evaluated in the future. The use of miR-126-3p for therapeutic or clinical purposes may face many challenges and controversies, including the specificity of its targets, specific mechanisms, doses, etc., which require further research. The expression level of miR-126-3p and its target factor in clinical patients and their predictive value for disease severity and prognosis need to be further explored. The subsequent development of clinical drugs that affect the expression of miR-126-3p may be a new research point.

## Figures and Tables

**Table 1:** Experimental operations of each group.

*Groups*	Operations
*Sham*	Only the right common carotid artery was exposed without ligation and hypoxia treatment.
*HI*	The right common carotid artery ligation and hypoxia treatment were performed to construct the HIBD model.
*i-NC+HI*	Intracerebroventricular injection of miR-126-3p mimics NC + constructing HIBD model.
*i+HI*	Intracerebroventricular injection of miR-126-3p mimics + constructing HIBD model.
*m-NC+HI*	Intracerebroventricular injection of miR-126-3p inhibitors NC + constructing HIBD model.
*m+HI*	Intracerebroventricular injection of miR-126-3p inhibitors + constructing HIBD model.
*m-NC+OE-NC+HI*	Intracerebroventricular injection of miR-126-3p inhibitors NC and LRP6 mimics NC + constructing HIBD model.
*m+OE-NC+HI*	Intracerebroventricular injection of miR-126-3p inhibitors and LRP6 mimics NC + constructing HIBD model.
*m+OE-LRP6+HI*	Intracerebroventricular injection of miR-126-3p inhibitors and LRP6 mimics + constructing HIBD model.
*m+OE-LRP6+PMA+HI*	Intracerebroventricular injection of miR-126-3p inhibitors, LRP6 mimics and PMA + constructing HIBD model.

## Abbreviations

ERK: Extracellular regulated protein kinases
HIBD: Hypoxic-ischemic brain damage
IL: Interleukin
LRP6: Low density lipoprotein receptor related protein 6
MAPK: Mitogen-activated protein kinase
miR: microRNA
PKC: Protein kinase C
TNF-α: Tumor necrosis factor-α
